# Safeguarding our soils

**DOI:** 10.1038/s41467-017-02070-6

**Published:** 2017-12-05

## Abstract

Alarming rates of soil degradation highlight the fragility of this precious resource and call into question our ability to achieve the UN’s Sustainable Development Goals by 2030. World Soil Day, December 5^th^, celebrates the importance of soils and calls for increased awareness and action to safeguard soil health and protect the prospects of a sustainable future.

Thoroughly mix crumbled rock, moisten with water, fold in pockets of air, and add generous helpings of dead, decaying and living organisms. While not particularly appetising, this is a recipe for healthy soil, a unique and complex matrix of minerals and organic matter, vital for sustaining life on Earth.

Healthy soils are essential for the growth of crops, filtration of water, functioning of ecosystems, and storage of vast amounts of carbon that would otherwise escape to the atmosphere. They are also under threat.

“Healthy soils are essential for the growth of crops, filtration of water, functioning of ecosystems, and storage of vast amounts of carbon that would otherwise escape to the atmosphere. They are also under threat.”

While continuously formed through the gradual erosion of rock, soil formation is vastly outpaced by the rate of degradation. Although the Hollywood blockbuster ‘The Martian’ featured Matt Damon ingeniously creating ‘soil’ in a day, back on Earth, and back in reality, 1 cm of soil can take up to 1,000 years to form, yet only days to erode.

Natural drivers of soil degradation are exacerbated through human activities such as agriculture, deforestation, and urban development. Soil is essentially a non-renewable resource, much like fossil fuels, and once destroyed it is, for all practical purposes, lost forever.

It is now estimated that as much as one-third of global soils are degraded, with up to 970 million tons of soil lost annually to erosion through poor management practices in Europe alone, and 24 billion tons lost globally^1^. For less developed nations where soil quality is already poor, the consequences of soil degradation are stark. Farming in Sub-Saharan Africa—where nearly one in four people remain undernourished^2^ and 38% of children under the age of 5 suffer from chronic malnutrition^3^—has led to further nutrient depletion and low cereal yields. Soil erosion further results in the loss of valuable carbon stores from the soil matrix, with one recent study estimating a cumulative global carbon debt of 133 billion tons since the dawn of agriculture^4^.

Despite its importance and vulnerability, broad awareness of the plight of our soils is poor. World Soil Day, celebrated annually since 2014, seeks to change this. Through outreach and educational activities, the Food and Agricultural Organisation (FAO) of the United Nations aims to draw attention to the importance of healthy soil and the need for the sustainable management of soil resources.

The sustainable use and management of soils will be intrinsic to the success of several of the United Nations’ Sustainable Development Goals (SDGs): food security (SDG 2), health (SDG 3), water quality (SDG 6), climate action (SDG 13) and biodiversity (SDG 15)^5^. The soil science research community is very much aware of the central role they have to play if a sustainable society is to be achieved by 2030^6^. Encouraging progress is being made, with considerable advances in increasing soil fertility through soil amendments practices such as the application of biochar—the use of pyrolised biomass, essentially charcoal, to enhance soil water and nutrient retention. Just this year, important insights have been made into the biogeochemistry and fertilisation properties of this sustainable approach^7,8^.

In parallel to soil fertility efforts, a new initiative has been launched to raise the awareness of soils as a potential climate mitigation tool. The proponents claim that sequestering atmospheric CO_2_ in the form of soil organic carbon by as little as 0.4% per year has the potential to offset anthropogenic emissions (www.4p1000.org). The FAO is further promoting the hidden potential of soil organic carbon, distilling the science into digestible information and thus facilitating the transfer of knowledge from the science domain to the policy arena^9^. The transfer of such knowledge is essential if potential solutions are to be successfully implemented.

Although many soil studies remain disciplinary and focussed on individual issues, soil scientists are making an effort to shift towards inter-disciplinary research, more holistic approaches in targeting multiple SDGs at once and engaging with land-users, businesses and policymakers. Integrated approaches and the adoption of sustainable land management practices will be required if global food security is to be achieved.

While there are many challenges to overcome before the SDGs 2030 target, the ambitious goals set out by the UN are not unattainable. Sustainable management practices can reverse soil degradation and restore productivity. Success in this regard has been realised in Ethiopia and Rwanda, where integrated soil fertility management practices have led to land restoration in recent years^10^. The SDGs represent an unprecedented opportunity to embrace sustainability on a global scale and educational initiatives such as World Soil Day are integral in setting events into motion that will ensure the well-being of future generations and of our planet. The recipe for the future very much needs to focus on safeguarding healthy soils to achieve sustainable success.
